# Interactivity mitigates the impact of working memory depletion on mental arithmetic performance

**DOI:** 10.1186/s41235-016-0027-2

**Published:** 2016-12-07

**Authors:** Frédéric Vallée-Tourangeau, Miroslav Sirota, Gaëlle Vallée-Tourangeau

**Affiliations:** 1 Department of Psychology, Kingston University, Kingston-upon-Thames, KT1 2EE UK; 2 Department of Psychology, University of Essex, Wivenhoe Park, Colchester, Essex CO4 3SQ UK; 3 Department of Management, Kingston University, Kingston-upon-Thames, KT2 7LB UK

**Keywords:** Interactivity, Mental arithmetic, Articulatory suppression, Working memory, Math anxiety, Systemic cognition

## Abstract

Doing long sums in the absence of complementary actions or artefacts is a multistep procedure that quickly taxes working memory; congesting the phonological loop further handicaps performance. In the experiment reported here, participants completed long sums either with hands down – the low interactivity condition – or by moving numbered tokens – the high interactivity condition – while they repeated “the” continuously, loading the phonological loop, or not. As expected, interactivity and articulatory suppression substantially affected performance; critically, the effect of articulatory suppression was stronger in the low than in the high interactivity condition. In addition, an independent measure of mathematics anxiety predicted the impact of articulatory suppression on performance only in the low (not high) interactivity condition. These findings suggest that interactivity augmented overall or systemic working memory resources and diminished the effect of mathematics anxiety, underscoring the importance of characterizing the properties of the system as it is configured by the dynamic agent-environment coupling

## Significance

This paper examines the role of interactivity in mental arithmetic. Interacting with physical resources that configure an arithmetic problem creates a shifting dynamic problem presentation; these dynamic changes in the problem’s appearance may actually perform some of the necessary computations, above and beyond the computations performed mentally by the agent. We examined the role of interactivity in mitigating the impact of depleted working memory resources (through articulatory suppression) and mathematics anxiety on mental arithmetic performance. Depleting working memory resources lead to a predictable decrement in mental arithmetic performance; however, the deterioration was less severe when participants could interact with physical artifacts that configured the problem. In addition, participants’ level of mathematics anxiety moderated the impact of working memory depletion on performance, but only in a low interactivity task environment. Mathematics anxiety and poor numeracy are comorbid. A consequence of mathematics anxiety is avoidance coping; phobias give rise to behavioral patterns that eliminate or reduce exposure to the aversive stimulus. As a consequence, there are fewer opportunities to engage with mathematics and receive positive feedback which would weaken avoidance coping. Interacting with the physical presentation of a problem results in the distribution of the computational and representational requirements of mental arithmetic across internal resources of the agent and environmental resources, augments the working memory capacity of the agent-environment system, enhances performance, and produces a more positive experience. Pedagogical interactive task environments may counteract avoidance coping and increase exposure to mathematics.

## Background

Different components of working memory are engaged in doing long sums without external aids or complementary actions (Raghubar, Barnes, & Hecht, 2010). The exact involvement of these components depends on the complexity of the arithmetic task, the presentation format and modality of presentation, as well as the agent’s level of mathematical competence (DeStefano & LeFevre, 2004). Take the task of adding a long series of single digit numbers presented visually all at once in a random pattern. Calculating the correct answer requires temporary storage and executive skills: interim totals are calculated and rehearsed sub-vocally, numbers tagged as having been added, others tagged as not, attention allocated to certain areas of the visual presentation or switched to others to identify what number or the easiest number to add next, and arithmetic knowledge retrieved from long-term memory to facilitate the identification of congenial subtotals. It is no surprise that loading the phonological loop in dual-task paradigms interferes with mental arithmetic that requires counting (Fürst & Hitch, 2000; Logie, Gilhooly, & Wynn, 1994).

### Complementary actions and interactivity

The role of working memory in mental arithmetic is traditionally established with an experimental procedure that limits or prevents participants from modifying the problem presentation in working out an answer. In order to create an unadulterated window onto the processes implicated in mental arithmetic and to permit the clinical precision of their segmentation, simple problems devoid of content are presented in a manner that cannot be modified by the agent. However, once released from the confines of the cognitive psychologist’s laboratory, mental arithmetic is often situated (Lave, 1988) and naturally supported by a range of complementary actions, such as pointing, which is used to guide attention and bind elements in a functional sequence (Carlson, Avraamides, Cary, & Strasberg, 2007; Kirsh, 1995). Gesturing can also facilitate and speed up the processing of information. Using a dual-task paradigm, Goldin-Meadow, Nusbaum, Kelly, and Wagner (2001) showed that participants perform better at a secondary memory task when gesturing while explaining how they solved a math problem (the primary task). Thus, gesturing appears to free up cognitive resources.

Imagine, again, adding a long series of single digit numbers, however, this time, the numbers do not configure a static visual presentation, but rather adorn the face of wooden tokens creating a malleable physical configuration which participants can modify as they work on the problem. The calculation unfolds along a spatiotemporal itinerary wrought by the agent’s actions. These actions modify the problem presentation and in doing so the problem is restructured: added numbers can be physically demarcated so they no longer exert attentional pull, and congenial interim totals (for example, 8 + 7) are identified and physically segregated, which shifts the affordances of what to do next and guides the agent to identify complementary subtotals (for example, 9 + 6) that interlock to create easy-to-remember provisional sums (for example, 30), to improve efficiency and reduce error. The reconfiguration of the problem guides, in part, the allocation of attentional resources and strategy selection (Vallée-Tourangeau, 2013); dynamic changes in the problem’s appearance may actually perform some of the necessary computations, above and beyond the computations performed mentally by the agent (see Rumelhart, Smolensky, McClelland, & Hinton, 1986). Interactivity creates an agent-environment system. On the one hand, interactivity reduces the demands on the agent’s working memory resources, but on the other, the system’s overall working memory resources are augmented.

### The present experiment

The present experiment employed a dual-task procedure to explore the impact of articulatory suppression in a mental arithmetic task. The task involved adding 11 single digit numbers presented either as a static configuration (a low interactivity condition) or as a set of number tokens that could be manipulated in calculating the answer (a high interactivity condition). Participants completed the task either with articulatory suppression—by repeating aloud “the” continuously—or without. Past research findings led us to expect poorer performance with articulatory suppression, but better performance with interactivity. We hypothesized that if interactivity transforms and augments the working memory resources of the agent-environment system, the impact of articulatory suppression on performance should be mitigated in the high interactivity condition, such as to result in an interaction between the two factors. Specifically, the performance advantage conferred by a high degree of interactivity should be greater with articulatory suppression than without.

Furthermore, we hypothesized a specific pattern of moderation effects. To this end, we profiled participants in terms of their: (i) basic arithmetic skills, (ii) level of mathematics anxiety, and (iii) executive function with an attention switching task. We used these concomitant variables to confirm that they moderate the impact of suppression on mental arithmetic, and that their impact on the low and high interactivity conditions would differ. We expected all three variables to moderate the impact of suppression primarily in the low interactivity condition; if a higher degree of interactivity augments overall or systemic working memory resources, then participants’ performance would be more resilient and the moderating properties of these factors might be attenuated.

## Methods

### Participants

In exchange for course credits, 52 Kingston University psychology undergraduate and postgraduate students (45 females) participated in the experiment (*M*_age_ = 21.8, *SD* = 4.0). We determined our sample size by an a priori set-up stopping rule of a sample size typically employed to detect an interactivity effect in previous experiments (for example, Allen & Vallée-Tourangeau, 2016; Vallée-Tourangeau, 2013). In terms of sensitivity, such a sample size (assuming a 2 × 2 repeated measures ANOVA, α = 0.05, 1 – β = 0.80) would enable us to detect a medium-to-small effect of *f* = 0.16 for the main effects and a correlation of *r* = 0.23 for the moderation effects (assuming α = 0.05, 1 – β = 0.80, and one-tailed test).

### Materials and measures

#### Arithmetic task

Participants were invited to add a series of 11 single digits. For each sum the digits were arrayed in a random cloud pattern, and were presented either on a sheet of A4 or as identically arranged wooden tokens. The sums in the low interactivity condition were presented with numbers that were 1 cm high and 0.5 cm wide, each number framed in a circle that was 2.5 cm in diameter; the tokens employed in the high interactivity condition were 2 cm in diameter and the digit inscribed on the tokens were 0.8 cm high and 0.4 cm wide. Participants were instructed to calculate the sum as quickly as they could and announce their answer to an experimenter. They did so either with their hands flat on the table top in front of them and were not allowed to use their fingers to count or point (low interactivity) or by moving the tokens about as they saw fit in producing an answer (high interactivity). Tracing paper templates of each of the sums presented in the low interactivity condition were created with holes for each number; these templates were used to arrange the spatial layout of the number tokens in the high interactivity condition. The experimenter placed a screen in front of the work surface to block the participants’ view and prevent them from adding the numbers before the tokens were all positioned; once the number tokens for a given sum were placed on the work surface, the paper template was lifted, the screen was removed and participants were invited to move the tokens to solve the problem. Latencies were measured from the time the screen was lifted and participants could see the display of tokens; in the low interactivity condition latencies were measured from the time the sheet of A4 was placed in front of the participant. Experimenters used a stopwatch to measure latency to solution.

Performance on this arithmetic task was measured in terms of accuracy – percentage correct and absolute calculation error – solution latencies, and efficiency. Participants’ efficiency at calculating the sums was measured as the ratio of their accuracy – percentage correct – over the resources invested in arriving at the answer. The latter was operationalized as the proportion of time taken to announce an answer out of the maximum time taken to announce an answer as indexed by the average latency of the slowest quartile. A ratio of 1 or greater indicated efficient performance, whereas a ratio below 1 indicated inefficient performance.

#### Mathematics Anxiety Scale

Participants completed a 25-item Mathematics Anxiety Scale-UK (Hunt, Clark-Carter, & Sheffield, 2011). The questionnaire invited participants to imagine how anxious they would feel in certain situations (1 = “not at all” and 5 = “very much”), such as “Working out how much your shopping bill comes to”.

#### Basic arithmetic skill

Basic arithmetic skill was measured by having participants complete as many of 45 simple expressions (such as 11 − 9 = ?) as they could in a 60-second period.

#### Executive function: shifting

The plus-minus task reported in Miyake, Friedman, Emerson, Witzki, and Howerter (2000) was employed to measure attention switching skills. With three different series of 30 double digit numbers, participants were instructed to add 3 to each in the first series, subtract 3 from each in the second series, and alternate between adding and subtracting 3 in the third series. The switching cost was the difference in completion time for the third series minus the average completion time for the first two.

### Procedure

Ten different sums of 11 single digits were created: none of the sums were the same and totals ranged from 57 to 80. From these, five were randomly selected and allocated to the low interactivity condition and the other five to the high interactivity condition for each participant. Participants completed these five sums twice within each level of interactivity: Once with articulatory suppression, once without. The design employed was a 2 (interactivity – low, high) × 2 (articulatory suppression – without, with) repeated measures.

The order of the four conditions for each participant was constructed as follows: one of the four conditions was randomly selected to be the first condition experienced by the participant. Once that first condition was identified, the order of the other three was determined by the following constraint: conditions with the same level of interactivity could not be presented in succession (for example, the two high interactivity conditions experienced consecutively). The first presentation of a condition with articulatory suppression was always preceded by a training task during which participants were asked to write successive subtractions of 3 starting from 100 for 1 minute while continuously repeating “the”. Participants experienced each interactivity condition twice: once with articulatory suppression, once without. In the articulatory suppression conditions if more than 2 seconds elapsed without participants engaging in the secondary task, they were prompted to comply with the task. Finally, the presentation of each condition was separated by the completion of one of three tasks: the basic arithmetic skill test, the Mathematics Anxiety Scale, or the attention switching task; the order of these three tasks was counterbalanced across participants. The Research Ethics Committee of Kingston University’s Faculty of Arts and Social Sciences conferred a favorable opinion on the research protocol.

## Results

### Mental arithmetic performance

#### Percentage correct

The mean percentages of correct additions in the four experimental conditions are reported in the top portion of Table 1. As expected, participants were better at providing correct answers in the absence of articulatory suppression; however, performance was generally better in the high interactivity condition. In addition, the decline in performance with articulatory suppression appeared steeper in the low interactivity condition. A 2 × 2 repeated measures analysis of variance (ANOVA) supported these impressions: the main effect of suppression was significant, *F*(1, 51) = 60.1, *p* <.001, *η*_*p*_^*2*^ = .54, as was the main effect of interactivity, *F*(1, 51) = 13.6, *p* = .001, *η*_*p*_^*2*^ = .21; the interaction was also significant, *F*(1, 51) = 4.06, *p* = .049, *η*_*p*_^*2*^ = .07. Post-hoc *t-*tests confirmed that while accuracy was higher in the high interactivity condition than in the low interactivity condition without suppression, the difference was not significant, *t*(51) = −1.75, *p* = .086; in turn, in the presence of suppression, accuracy was significantly higher in the high interactivity condition than in the low interactivity condition, *t*(51) = −4.21, *p* <.001.

**Table 1:** Mean (and SD) for the percentage correct, latency (s), and efficiency ratio for the five sums

	Articulatory suppression			
	Without		With	
	Percentage correct			
Interactivity	*M*	*SD*	*M*	*SD*
Low	56.0%	27.7%	26.9%	31.7%
High	63.5%	28.2%	44.6%	29.5%
	Latencies			
	*M*	*SD*	*M*	*SD*
Low	38.1	16.4	41.5	21.2
High	37.4	14.8	51.2	27.2
	Efficiency ratio			
	*M*	*SD*	*M*	*SD*
Low	1.20	0.99	0.68	0.95
High	1.16	0.70	0.99	0.84

#### Absolute calculation error

**Fig. 1:**
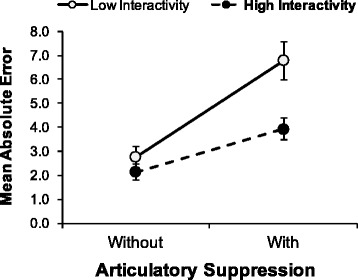
Mean absolute calculation error (with standard errors)

The mean absolute calculation errors in the four conditions are plotted in Fig. 1. These data illustrate a substantial effect of suppression, with larger deviations from the correct answers recorded with suppression than without. Errors were generally smaller in the high interactivity condition and, more importantly, articulatory suppression appeared not to have as dramatic an impact on calculation accuracy in the high interactivity condition. In a 2 × 2 repeated measures ANOVA the main effects of suppression, *F*(1, 51) = 36.2, *p* <.001, *η*_*p*_^*2*^ = .42 and interactivity, *F*(1, 51) = 9.69, *p* = .003, *η*_*p*_^*2*^ = .16, were significant, as was the interaction, *F*(1, 51) = 6.02, *p* = .018, *η*_*p*_^*2*^ = .11. Follow-up *t*-tests revealed that high interactivity yielded somewhat lower but not significantly lower absolute calculation errors than low interactivity in the absence of suppression, *t*(51) = 1.31, *p* = .197. In turn, calculation errors were significantly lower in the high interactivity condition with articulatory suppression, *t*(51) = 3.18, *p* = .003.

#### Solution latency

The mean solution latencies are reported in the middle portion of Table 1. In the absence of articulatory suppression, solution latencies were similar in the low and high interactivity conditions. Although the participants were generally slower with articulatory suppression, they were slowest in the high interactivity condition. A 2 × 2 repeated measures ANOVA revealed a significant main effect of suppression, *F*(1, 51) = 16.4, *p* <.001, *η*_*p*_^*2*^ = .25, a significant main effect of interactivity, *F*(1, 51) = 11.5, *p* <.001, *η*_*p*_^*2*^ = .19, as well as a significant interaction, *F*(1, 51) = 18.6, *p* <.001, *η*_*p*_^*2*^ = .27. In light of the significant interaction, subsequent *t* tests confirmed that latencies were similar in the two interactivity conditions in the absence of suppression, *t*(51) = 0.51, *p* = .613, but were significantly slower in the high interactivity condition, *t*(51) = −4.69, *p* <.001.

#### Efficiency ratio

The mean efficiency ratios are reported in the bottom portion of Table 1: participants were much more efficient in the absence of articulatory suppression, and efficiency declined sharply with suppression. However, in the high interactivity condition, participants’ efficiency ratio remained good even with articulatory suppression. A 2 × 2 repeated measures ANOVA supported these impressions: the main effect of suppression was significant, *F*(1, 51) = 32.8, *p* <.001, *η*_*p*_^*2*^ = .39, but the main effect of interactivity was not, *F*(1, 51) = 2.61, *p* = .113, *η*_*p*_^*2*^ = .05; however, the interaction between suppression and level of interactivity was significant, *F*(1, 51) = 7.47, *p* = .009, *η*_*p*_^*2*^ = .13. Post-hoc *t* tests indicated that participants’ efficiency at completing the task was not influenced by interactivity in the absence of suppression, *t*(51) = 0.43, *p* = .666; however, participants were significantly more efficient completing the sums in the high interactivity condition with articulatory suppression, *t*(51) = −2.91, *p* = .005.

### Moderators of the impact of suppression on calculation error

To test our moderation hypotheses we conducted a moderation analysis for within-subject design using ordinary least square regression with difference scores, as proposed by Judd, Kenny, and McClelland (2001). This was a preferred solution for the current experiment with a sample size that is not optimal for a multilevel modelling. Judd et al. (2001) suggested that the moderation in within-subject designs occurs when a concomitant variable (for example, basic arithmetic skill, math anxiety level) predicts differences in performance between two conditions. Thus we determined how these variables moderated the difference in performance with and without articulatory suppression within each level of interactivity, and then by collapsing the level of interactivity. Table 2 reports the correlations between each of the concomitant variables and the difference in absolute calculation errors between the condition with articulatory suppression and the condition without, when interactivity level is low and high, and when collapsing over the two levels of interactivity (*df* = 50 for all correlation coefficients).

**Table 2:** Correlation between increase in absolute calculation error with suppression and the three concomitant variables

	Interactivity condition		Overall
	Low	High	
BAS	−.28*	−.27	−.37**
MAS	.41**	.01	.34*
SWITCH	−.01	.04	.01

*BAS* basic arithmetic skill, *MAS* Mathematics Anxiety Scale, *SWITCH* attention switching score. *p <.05, **p <.01

#### Basic arithmetic skill

Overall, the increase in absolute calculation error when collapsing across interactivity conditions was moderated by basic arithmetic skills, *r* = −.37, *p* = .007; that is, the higher the participants’ arithmetic skill, the smaller the increase in calculation error with articulatory suppression. This relationship was observed in both the low, *r* = −.28, *p* = .042, and high interactivity condition, *r* = −.27, *p* = .056; the difference in the magnitude of the correlations between the two interactivity conditions was not significant, *Z* = 0.053, *p* = .957.

#### Math anxiety

When collapsing the data over both interactivity conditions, levels of mathematic anxiety moderated the impact of articulatory suppression, *r* = .34, *p* = .014; that is, the higher the level of math anxiety, the higher the increase in calculation error with articulatory suppression. However, this overall pattern obscures a more interesting pattern across levels of interactivity. Thus, in the low interactivity condition math anxiety was a significant moderator of the increase in error with suppression, *r* = .41, *p* = .003, but not in the high interactivity condition, *r* = .01, *p* = .951; the correlation was significantly more positive in the low interactivity than in the high interactivity condition, *Z* = 2.107, *p* = .035.

#### Attention switching

As the correlation coefficients reported in the bottom row of Table 2 indicate, scores on the attention switching test did not moderate the increase in calculation error with articulatory suppression.

## Discussion

This experiment explored how interactivity could mitigate the impact of a reduction in working memory resources through articulatory suppression on mental arithmetic performance. While mental arithmetic performance was always poorer with articulatory suppression, deterioration was always significantly greater when participants completed the sums with their hands palm down on the table top. Crucially, interactivity attenuated the impact of a secondary task that taxed the phonological loop, which reduced participants’ ability to rehearse interim totals or plan counting strategies sub-vocally. The possibility of restructuring the physical problem presentation over the course of the calculation ensured that the participants could reconfigure the environment in a manner that compensated for the reduction in internal working memory capacity. The combined resources of the agent coupled to a malleable environment in the high interactivity condition did not soak up completely the resources depletion caused by articulatory suppression since performance was affected by the secondary task, but it was sufficiently robust to ensure efficient calculations.

It is important to acknowledge that different materials were employed in the low and high interactivity conditions. The size of the numbers was a little bigger and the number-background contrast was a little sharper in the low interactivity than in the high interactivity condition. It is not clear on the basis of the present data whether these differences acted to reduce the benefit of interactivity, since in the absence of suppression the participants’ accuracy only improved marginally in the high interactivity condition. Certainly, previous experiments that have used the same material in both low and high interactivity conditions have reported substantial effects of interactivity on performance (for example, Allen & Vallée-Tourangeau, 2016; Carlson et al., 2007); although we note that in the study of Carlson et al. the performance improvement in a high interactivity condition was marginal with sums that were of the same length as those employed in the present experiment (viz., 11 numbers long; see their Fig. 2, p. 753), but much more pronounced with longer sums. It is possible that the addition task was insufficiently demanding in the absence of suppression for the benefit of interactivity to manifest significantly in terms of improved accuracy and reduced calculation error. As for the minor differences in materials across condition, the perceptual information probably varied with head movements and posture adjustments as participants solved the additions. More importantly, the physical configuration of the problem changed in the high interactivity condition: thus, participants’ actions wrought changes in the physical configuration of the problem, changes that are contingent on the preceding state of the problem. We propose that a higher degree of interactivity elevates performance in part because it creates a dynamic problem configuration that not only lightens the load, but also leads to the creation of congenial interim sums and to physically segregated categories of elements (such as those counted, those yet to be counted). Thus different arithmetic strategies can be enacted with a malleable problem presentation (Vallée-Tourangeau, 2013).

Keeping hands still in a task that naturally scaffolds on gestures and the use of fingers to anchor attention and binds elements to their functional roles (Carlson et al., 2007) probably requires some inhibitory control that, in turn, requires some executive capacity. However, it is important to note that Goldin-Meadow et al. (2001) found no difference in performance when participants were instructed not to gesture and when they naturally chose not to gesture in a gesture-allowed context. Thus, it is unlikely that preventing participants from using their hands co-opted executive capacity in a manner that offered a substantial explanation of the inferior arithmetic performance in the low interactivity condition. Pointing alone is a complementary action (Kirsh, 1995) that elevates performance in a variety of counting tasks (for example, Neth & Payne, 2011; Guthrie, Mayer, & Vallée-Tourangeau, 2014). A higher degree of interactivity elevates performance; however, it is not possible to apportion the relative contribution of the changing problem configuration and complementary actions (such as pointing) to the performance improvement with the procedure employed in the present experiment. Finally, the present experiment cannot determine the contribution of embodiment in the performance improvement in the high interactivity condition. A condition where participants direct an experimenter to move the tokens on their behalf while remaining still themselves, may help us better understand the role of embodiment. Such a control condition would not eliminate the participants’ sense of agency since they could still create a dynamic problem presentation, and they would still be coupled to the environment, albeit indirectly. To eliminate the agent-environment coupling as well as embodiment would require a yoked control condition where passive participants either watch a video of a successful participant in a high interactivity condition or are presented with a still image of the final problem configuration of a participant who announced the correct answer. It is plausible to conjecture that such yoked participants may benefit from the changes in the initial physical configuration of the problem, recognize some or all of the emerging groupings, and see how these groupings can be combined to arrive at the answer. However, a participant’s action and engagement with the task in the high interactivity condition may create a series of changes to the problem configuration that chart a singular and contingent spatiotemporal trajectory that benefits the participant qua agent much more than a passive observer. These remain important empirical questions.

Accuracy in the high interactivity conditions dropped by 20% with articulatory suppression, and latency increased by nearly 14 seconds on average (a 37% increase in latency). In contrast, latency across the low interactivity conditions increased by 3.4 seconds on average with articulatory suppression (a 9% increase in latency). At first, the latency data might suggest participants did not fully engage with the secondary task in the low interactivity condition, yet accuracy was down by 30% and absolute calculation errors were four times as large with articulatory suppression in the low interactivity condition (see Fig. 1). Rather, what these relatively short latencies indicate was that the task was very hard in the low interactivity condition with articulatory suppression: participants abandoned calculations more quickly in the low interactivity condition than in the high interactivity condition and were more likely to guess the answer. It is interesting to note that participants’ level of mathematics anxiety was a significant moderator of the impact of suppression on calculation error, but only in the low interactivity condition. This suggests that participants anxious about math might have guessed more in the low interactivity condition, reducing problem latency but also increasing error. This pattern has been previously reported in the math anxiety literature (for example, Ashcraft & Krause, 2007). Thus, in this simple arithmetic task, the reduction of internal working memory capacity through articulatory suppression had its most deleterious effect on participants with higher levels of math anxiety.

Participants’ basic arithmetic skills moderated the impact of articulatory suppression, in both the low and high interactivity conditions. In turn, math anxiety moderated the impact of suppression only in the low interactivity condition; this finding suggests that a higher degree of interactivity produced more resilient performance irrespective of differences in anxiety. Finally, the attention switching scores did not moderate the impact of suppression on performance either in the low or high interactivity condition. To the extent that this task gauges participants’ ability to switch their attention, we expected these scores to correlate positively with changes in performance as a function of suppression; they did not. A more precise measure of attention switching, perhaps using an automated task or a composite score from different attention switching tasks, might offer a more informative window on how switching skills might moderate the influence of articulatory suppression on mental arithmetic.

## Conclusions

Overall, we consider these moderation patterns to be quite revealing. Psychometric efforts to unveil the cognitive capacities and dispositions subserving performance in a domain must be interpreted relative to a context of reasoning. In the experiment reported here, the context varied in terms of the degree of interactivity it afforded and the cognitive resources that could be deployed on the primary task. Math anxiety may be an important moderator of mental arithmetic performance (Ashcraft, 2002). There is much evidence to suggest that math anxiety is associated with lower working memory capacity (Passolunghi, Caviola, De Agostini, Perin, & Mammarella, 2016); working memory resources may be further compromised by intrusive math-averse ideation while performing a math task (Ashcraft & Krause, 2007; Carey, Hill, Devine, & Szücs, 2016; Ramirez, Gunderson, Levine, & Beilock, 2013). Our findings demonstrate that allowing participants to interact with a malleable problem presentation attenuates the impact of math anxiety on performance. A systemic perspective on cognition seeks to describe cognitive products and processes of a system configured by the dynamic coupling of an agent and his or her physical environment (Vallée-Tourangeau, Abadie, & Vallée-Tourangeau, 2015). In the present mental arithmetic task, interacting with the physical problem presentation transformed an agent’s ability to solve these problems. The resulting performance invites a characterization of the cognitive capacities of the system rather than of the agent.
